# Sex-Related Differences in Cardiovascular Disease Risk Profile in Children and Adolescents with Type 1 Diabetes

**DOI:** 10.3390/ijms221910192

**Published:** 2021-09-22

**Authors:** Darja Smigoc Schweiger, Tadej Battelino, Urh Groselj

**Affiliations:** 1 Faculty of Medicine, University of Ljubljana, Vrazov trg 2, 1000 Ljubljana, Slovenia; darja.smigoc@kclj.si (D.S.S.); tadej.battelino@mf.uni-lj.si (T.B.); 2 Department of Endocrinology, Diabetes and Metabolic Diseases, University Children’s Hospital, University Medical Centre Ljubljana, Bohoriceva 20, 1000 Ljubljana, Slovenia; 3 Department of Cardiovascular Medicine, School of Medicine, Stanford University, 870 Quarry Road, Stanford, CA 94305, USA

**Keywords:** sex, gender, cardiovascular risk factors, type 1 diabetes, children, adolescents, blood pressure, hyperglycemia, dyslipidemia, inflammation, smoking

## Abstract

Cardiovascular disease (CVD) is the primary cause of higher and earlier morbidity and mortality in people with type 1 diabetes (T1D) compared to people without diabetes. In addition, women with T1D are at an even higher relative risk for CVD than men. However, the underlying pathophysiology is not well understood. Atherosclerotic changes are known to progress early in life among people with T1D, yet it is less clear when excess CVD risk begins in females with T1D. This review explores the prevalence of classical CVD risk factors (such as glycemic control, hypertension, dyslipidemia, obesity, albuminuria, smoking, diet, physical inactivity), as well as of novel biomarkers (such as chronic inflammation), in children and adolescents with T1D with particular regard to sex-related differences in risk profile. We also summarize gaps where further research and clearer clinical guidance are needed to better address this issue. Considering that girls with T1D might have a more adverse CVD risk profile than boys, the early identification of and sex-specific intervention in T1D would have the potential to reduce later CVD morbidity and excess mortality in females with T1D. To conclude, based on an extensive review of the existing literature, we found a clear difference between boys and girls with T1D in the presence of individual CVD risk factors as well as in overall CVD risk profiles; the girls were on the whole more impacted.

## 1. Introduction

Cardiovascular diseases (CVD) are the leading cause of morbidity and mortality in persons with type 1 diabetes (T1D) [1], and it is more frequent in this population than in individuals without diabetes [2,3]. Indeed, people with early-onset T1D have up to 30 times higher probability of severe cardiovascular outcomes as compared to healthy individuals. Overall, those diagnosed with T1D before 10 years of age die an average of 16 years earlier, and the lives of those diagnosed at age 26–30 years are shortened by an average of ten years [4]. The atherosclerosis process is accelerated in people with T1D, and it begins early in childhood at the endothelial level [5,6,7,8,9]. Few data exist on the impact of CVD risk factors in pediatric subjects with T1D, while the early vascular damage and progression of atherosclerosis have been clearly described through intima–media thickness of the carotids and aorta starting from the very first years after the onset [10,11].

Hyperglycemia is considered the primary mediator of atherosclerosis in T1D. In the Diabetes Control and Complications Trial/Epidemiology of Diabetes Interventions and Complications (DCCT/EDIC) cohort with T1D, hyperglycemia was a major factor responsible for the increased CVD, second only to age. For every 1% increase in mean HbA1c, the risk for any CVD increased by 31% [12]. Further epidemiological analysis described the risk of any CVD associated with a 1 percentage point increase in the mean HbA1c as equivalent to the risk associated with 4.3 additional years of age or 5.6 additional years duration of T1D [13]. Several other modifiable risk factors such as hypertension, dyslipidemia, obesity, insulin resistance, lack of exercise, smoking, depression, and autonomic dysfunction further influence the CVD risk [14,15]. In addition, various nonmodifiable genetic factors, such as apolipoprotein E (ApoE) genetic variants, were also shown to modify the CVD risk [16,17].

The mortality ratio for CVD has been reported to be increased tenfold in men with T1D and 40-fold in women with T1D before the age of 40 years [18,19]. This might reflect the higher prevalence of CVD risk factors in females with T1D as compared to males or healthy women [5]. In a study by Brown et al., HbA1c levels, total cholesterol (Tc), low-density lipoprotein cholesterol (LDL-c), body mass index (BMI), and blood pressure (BP) were higher in females than in males, allowing the authors to conclude that, independently of other risk factors, female sex appears to modulate the impact of other mediators on CVD risk in a more atherogenic profile [20]. However, it is less clear when excess CVD risk begins in females with T1D.

Considering that girls with T1D might have a more adverse CVD risk profile than boys, early identification and sex-specific intervention would have the potential to reduce later CVD morbidity and mortality excess in females with T1D.

This review explores the prevalence of classical CVD risk factors (such as hyperglycemia, high blood pressure, dyslipidemia, obesity, albuminuria, smoking, diet, physical inactivity, and socioeconomic status), as well as of other biomarkers (such as chronic inflammation and insulin resistance), in children and adolescents with T1D with particular regard to sex-related differences in risk profile. We also summarize gaps wherein further research and clearer clinical guidance are needed to better address this issue. For this purpose, systematic searches of the literature were performed from inception up to 1 September 2021. We searched for published data using the keywords “Type 1 diabetes”, “Cardiovascular risk factors”, “Sex”, “Gender”, “Women”, “Children”, “Adolescents”, “Atherosclerosis”, “Sex hormones”, “Estrogens”, “Endothelial dysfunction”, “Oxidative stress”, “ Hyperglycemia”, “Blood pressure”, “Albuminuria”, “Dyslipidemia ”, “ Inflammation ”, “Obesity”, “Smoking ”, “Physical inactivity”, “Diet”, “Eating disorders”, “Depression”, “Diabetes distress”, “Health-related quality of life”, “Diabetes management”, “Multiple cardiovascular risk factors”, “Recommendations”, “Screening”, and “Prevention” in the English language in the PubMed, Mendeley, and Cochrane databases.

## 2. Cardiovascular Risk Factors in Type 1 Diabetes Youths and the Role of Sex

### 2.1. Hormonal Factors

**Figure 1:**
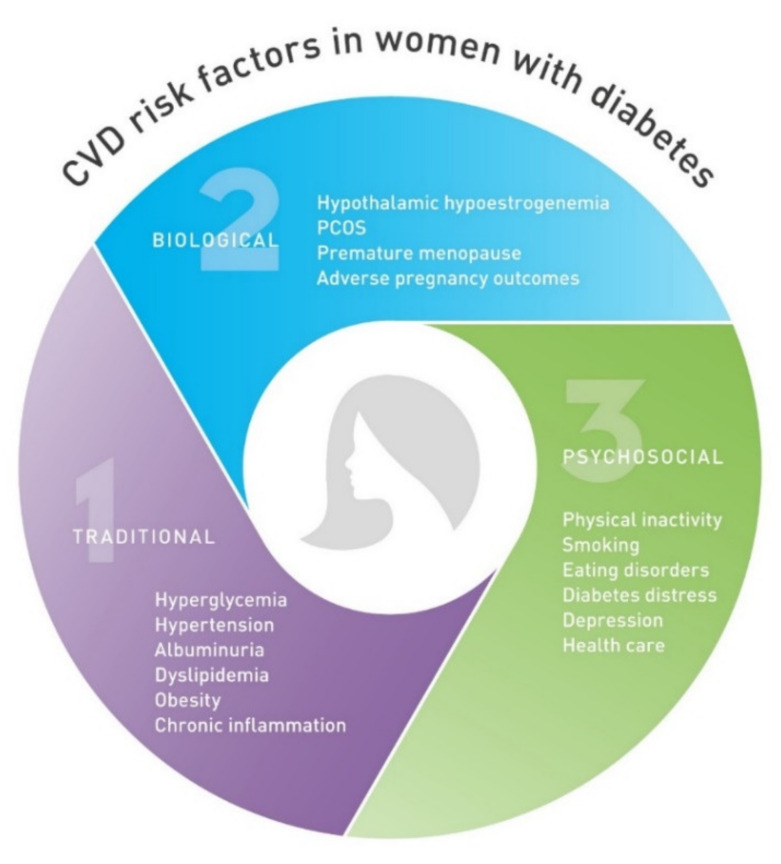
Cardiovascular disease (CVD) risk factors unique to women with diabetes. CVD, cardiovascular disease; PCOS, polycystic ovary syndrome.

Given the delayed onset of CVD in woman from the general population, premenopausal women seem to be protected from CVD compared with men of similar age [21]. These differences have been attributed to biological differences, which include sex chromosomes and hormonal status, as well as gender differences in behavioral and sociocultural variables [22]. Considering that risk of CVD increases as estrogen levels decline following menopause, estrogens appear to be protective against CVD [23]. Estrogens exhibit protective cardiovascular and metabolic effects through several pathophysiological pathways. By binding to estrogen receptors alpha, beta, and G-protein-coupled estrogen receptor, they confer both genomic and immediate nongenomic actions. Estrogen receptor expression in arteries is higher in women than in men but decreases with menopause [24]. Nongenomic signaling can result in rapid endothelial nitric oxide synthase (eNOS) activation and nitric oxide (NO) production, which facilitate vasodilation. [25]. Estrogen exerts antiproliferative effects on vascular smooth muscle cells (VSMC), promotes reendothelialization, reduces the proinflammatory activation of vascular endothelial cells and regulates production of reactive oxygen species (ROS) [26]. In blood, estrogen alters the lipoprotein profile, which increases the amount of high-density lipoprotein cholesterol (HDL-c) and inhibits LDL-c oxidation, which in turn reduces oxidized LDL-c accumulation in arterial wall [27]. In the adipose tissue, estrogen influences adipogenesis and adipose tissue localization, metabolism, and function, resulting in increased visceral adipose tissue and leading to visceral obesity and subsequent metabolic syndrome [28]. In addition to traditional risk factors, there are risk factors unique to women that contribute to CVD development. Adverse pregnancy outcomes such as pre-eclampsia, hypertensive disorders of pregnancy, gestational diabetes, preterm delivery, intrauterine growth restriction, and miscarriage have been associated with an increased CVD risk [29]. Even in normal pregnancies. women with five or more live births were at greater risk of coronary heart disease [30]. On the other hand, lactation has been shown to be associated with lower maternal cardiovascular risk [31]. Other risk-enhancing factors specific to women include premature menarche, premature ovarian failure, and polycystic ovary syndrome [32]. Although traditional CVD risk factors including elevated blood pressure, smoking, overweight and obesity, diabetes, and elevated cholesterol are relevant to both sexes, there are differences in their potency [33]. Diabetes confers a higher degree of risk for CVD in women than in men. Therefore, sex-related protection from CVD is lost in women with diabetes. Women with T1D have a roughly 40% greater excess risk of all-cause mortality, and twice the excess risk of fatal and nonfatal vascular events, compared with men with T1D [34]. The cause of this higher relative risk in women with T1D compared to that in men remains unclear. The mechanisms investigated so far are linked to both biological and psychosocial factors as well as management of diabetes and CVD risk factors [35] (Figure 1). Biological mechanisms are mostly linked to disturbance in the hypothalamic–pituitary–ovarian axis. Adolescents with T1D have been shown to have lower levels of estradiol [36] compared to nondiabetic control women. Among premenopausal women with diabetes, hypothalamic hypoestrogenism was more prevalent and associated with angiographic coronary artery disease [37]. Hormonal disbalance in women with T1D may therefore contribute to more atherogenic lipid profile, insulin resistance, higher inflammation, and loss of vasoprotective effect.

### 2.2. Hyperglycemia

**Figure 2:**
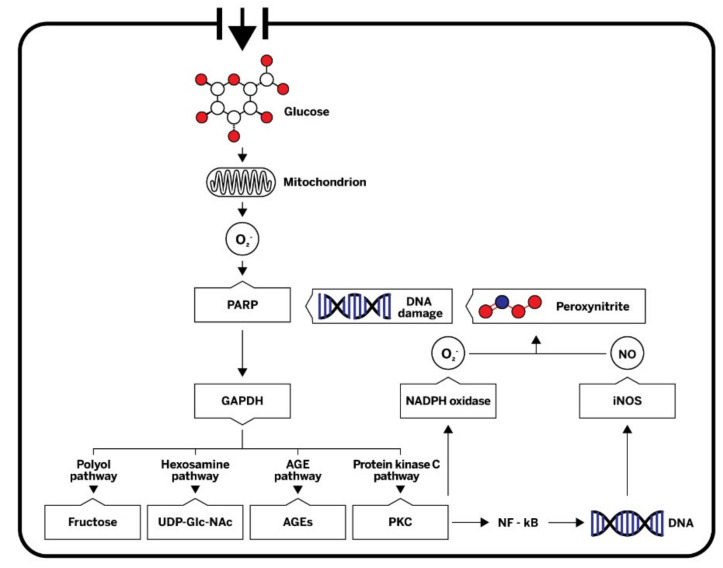
A simplified scheme of mechanisms by which hyperglycemia induces metabolic derangement in endothelial cells. Hyperglycemia-induced overproduction of mitochondrial superoxide activates four major pathways of hyperglycemic damage by inhibiting GAPDH. PKC and NF-κB are also activated, affecting nitric oxide (NO) production and inflammatory response. NO can react with superoxide, leading to the formation of the highly reactive peroxynitrite, which in turn damages DNA. Single DNA strand breaks stimulate the activation of PARP, thereby resulting in decreased activity of GAPDH. O_2_^−^, superoxide; PARP, poly (ADP-ribose) polymerase; GAPDH, glyceraldehyde-3-phosphate dehydrogenase; UDP-GlcNAc, uridine diphosphate N-acetylglucosamine; AEGs, advanced glycation end-products; PKC, protein kinase C; NADPH, nicotinamide adenine dinucleotide phosphate; DNA, deoxyribonucleic acid, NF-κB, nuclear factor kappa B; iNOS, inducible nitric oxide synthase; NO, nitric oxide.

High blood glucose levels impact atherosclerosis development by a diverse mechanism. Dysfunction of the vascular endothelium occurs early during atherogenesis, and hyperglycemia has been identified as one of the key causes. The uptake of glucose from the systemic circulation into vascular endothelial cell is mediated by glucose transporter 1 (GLUT-1) in an insulin-independent manner [38]. There are four main potential mechanisms for hyperglycemia-induced metabolic derangement in endothelial cells: increased polyol pathway, increased advanced glycation end-product (AGE) formation, activation of protein kinase C (PKC) isoforms, and increased hexosamine pathway. All four pathways are activated by common hyperglycemia-induced superoxide overproduction by the mitochondrial electron-transport chain (Figure 2) [39]. Overproduction of mitochondrial superoxide stimulates poly-ADP-ribose polymerase (PARP), which decreases activity of glycolytic enzyme glyceraldehyde-3-phosphate dehydrogenase (GAPDH). Consequently, metabolites from glycolysis are shunted into pathways of glucose overutilization. Activation of PKC contributes to endothelial dysfunction by activation of nuclear factor kappa B (NF-κB) and nicotinamide adenine dinucleotide phosphate (NADPH) oxidase, affecting NO production and inflammatory response. Hyperglycemia-induced, PKC-dependent activation of NADPH oxidase in endothelial cells is considered to be one of the major sources of ROS overproduction, such as superoxide [40]. Excess superoxide contributes to endothelial dysfunction primarily because of rapid oxidative inactivation of vasoprotective NO [41]. Activation of NF-κB transcription factors in endothelial cells leads to the expression of adhesion molecules, which facilitate monocyte and T-cell adhesion, and induces secretion of inflammatory mediators. In addition, inducible nitric oxide synthase (iNOS) is heavily upregulated by NF-κB [42]. After induction of iNOS elevated levels of NO can react with superoxide, leading to the formation of atherogenic peroxynitrite. Highly reactive peroxynitrite can cause various kinds of cellular damage, including single DNA strand breaks, which can stimulate PARP [43]. Hyperglycemia has a similar effect in VSMC, leading to proliferative and secretory properties that are involved in the development of atherosclerosis [44]. In addition, glycosylation of the apolipoprotein B in LDL-c leads to impaired uptake by the LDL receptor and decreased LDL-c plasma clearance. Furthermore, glycated LDL-c has increased susceptibility towards oxidation, which is considered a crucial step in atherogenesis [45]. Hyperglycemia was associated with preclinical atherosclerotic CVD in studies that included surrogate measures such as carotid intima–media thickness (CIMT) [46], arterial stiffness [47], and endothelial function [48] in T1D.

While numerous studies have established the link between inadequately controlled blood glucose level and microvascular complications of diabetes, the relationship between elevated blood glucose and macrovascular outcomes appears to be less straightforward [49]. Interventional clinical trials have investigated the effect of intensive treatment of hyperglycemia on cardiovascular risk reduction in T1D. A major T1D prospective, randomized clinical trial, the DCCT, and its observational follow-up, the EDIC study, showed long-term cardiovascular benefits of intensive glycemic control [50]. Intensive glycemic control substantially reduced patients’ risk of microvascular complications compared to conventional therapy [51]. Remarkably, the benefits of intensive glycemic control persisted throughout several decades of follow-up, showing a 30% reduction in CVD events [52] and a modest reduction in all-cause mortality [53] in the former intensive treatment group compared to the former conventional treatment group. Importantly, these findings showed that intensive treatment of hyperglycemia initiated early in individuals with T1D resulted in long-term benefits for CVD reduction. The long-term effect of exposure to high glucose levels for a certain period of time on diabetic cardiovascular complications is known as metabolic memory. This effect was statistically explained by the difference in HbA1c [54]. A study in the DCCT cohort using multivariate models found that HbA1c was an extremely important risk factor for major cardiovascular events, second only to age [12].

In contrast to DCCT, a prospective follow-up of observational cohorts showed mixed results in regard to an association between glycemic control and CVD risk. In the Wisconsin Epidemiologic Study of Diabetic Retinopathy, HbA1c was not significantly associated with angina or myocardial infarction [55]. In the Pittsburgh Epidemiology of Diabetes Complications (EDC) study, glycemia did not predict incident coronary artery disease [56], although more recent analyses did show an association between glycemic control and 25-year CVD incidence [57]. A study from the Swedish National Diabetes Register reported that individuals with T1D and HbA1c levels of 6.9% or lower still had a risk of death from CVD that was twice as high as the risk in the general population. The risk was several times higher among individuals with higher HbA1c levels [1]. Nevertheless, HbA1c, as an established indicator of blood glucose control, was demonstrated as a reliable risk factor of all-cause and CVD mortality in both diabetics and nondiabetics [58].

Because of metabolic memory, good metabolic control has beneficial effects even in the early stages of T1D to reduce CVD risk later in life [59]. However, the DCCT showed worse metabolic control in adolescents compared to adults [60]. Glycemic control often deteriorates during adolescence, reaching the highest values in late adolescence followed by a gradual improvement in young adulthood. In a large, prospective, 10-year, longitudinal study from the Germany/Austrian Diabetes Prospective Follow-up Registry (DPV), a strong age dependency was seen. In children <10 years old, the median HbA1c was 7.6%, and in patients aged 11–20 years, median HbA1c peaked to 8.7% at ages 16–18 years and then declined to 7.8% at the age of 20 [61]. In the cross-sectional data from the T1D Exchange Clinic Registry (T1D Exchange), a high proportion of adolescents did not reach targets for metabolic control. A worsening in glycemic control was seen at 13–17 years of age, with the mean HbA1c level of 9.0% being only slightly lower than the 9.5% seen in adolescents at the start of the DCCT [62]. Another large retrospective cohort study from T1D Exchange reported an increase in HbA1c levels from ages 8 to 16 years, stable levels between the ages of 16 and 18 (when HbA1c was 8.9%), and then a gradual decline during the young adult years to an HbA1c level of 8.2% [63].

Data suggest that females have worse metabolic control than males, especially during adolescence (Table 1). In a DPV study population, including 27,035 children, adolescents, and young adults, there was a significant association between metabolic control and sex. In all age groups, girls had a higher median HbA1c level (7.81%) than boys (7.75%) with a mean difference 0.1% (*p* < 0.0001) [61]. In a Slovenian registry, which included 886 case subjects from 0 to 17.99 years of age at diagnosis, females had on average 1.02 times higher values of HbA1c (*p* = 0.009), a higher probability of suboptimal metabolic control (*p* = 0.02), and a statistically nonsignificant higher probability of poor metabolic control compared to males [64]. A single-center, cross-sectional study evaluated sex-related differences in the CVD risk profile of 300 adolescents with T1D and 100 nondiabetic adolescents. Mean age was 15 years. Compared with boys with T1D, girls with the disease had significantly increased mean HbA1c (9.1% vs. 8.7%, respectively) [20]. Longitudinal data from 4430 boys and 3590 girls from the Swedish pediatric diabetes quality registry showed that girls had higher HbA1c than boys during follow-up, with the most obvious difference in the oldest age group [65]. In another Swedish longitudinal study with 1543 children and adolescents, the sex-related difference, with higher mean values of HbA1c in girls, persisted during adolescence but not during early adulthood [66]. An age-dependent difference in metabolic control between males and females was also shown in data obtained from 874 participants from the ages of 14 to 30 registered in the Norwegian Diabetes Register, with females having worse glycemic control, especially during adolescence. Females in their late teens had the highest median HbA1c, but the difference did not persist in young adulthood [67]. On the other hand, European data for 324501 people gathered in regional or national registries showed a small but persistent difference between both sexes in all age groups, including young adults [68]. The difference was also observed in a cross-sectional, single-center, observational study of 300 young adults with T1D and a mean age of 24.7 years. Women experienced less favorable results regarding glycemic control (HbA1c 8.4% ± 1.3% women vs. 8.1% ± 1.3% in men; *p* = 0.02). However, women were found to have higher HbA1c and glucose variability compared with men only when treated with multiple daily injections (MDI) and not when treated with continuous subcutaneous insulin infusion (CSII), suggesting alternative insulin delivery methods should be considered to increase diabetes management adherence [69].

Sex-related differences in glycemic control in adolescence may be due to sex-specific hormonal changes, insulin resistance, and increasing insulin requirements during pu-berty. In young subjects with T1D, insulin sensitivity positively correlated to growth hor-mone concentration and BMI [104]. It has been shown that healthy girls were less insu-lin-sensitive than boys but compensated their decreased sensitivity with increased insulin secretion [105]. In line with this finding, significantly higher insulin doses along with higher HbA1c were shown in Iranian pubertal girls [106]; however, there were no differ-ences in insulin doses between girls and boys in a study by Brown et al. [20]. In addition to hormonal factors, adherence to treatment and lifestyle recommendations usually de-creases during adolescence [107]. Eating disorders, depression, and peer relations were related to higher HbA1c among girls [108].

Because of metabolic memory, good glycemic control in adolescence is associated with lower risk of CVD complications later in life [59]. Guidelines released by the Interna-tional Society for Pediatric and Adolescent Diabetes (ISPAD) [109] and guidelines pub-lished by the American Diabetes Association (ADA) [110] state that for children, adoles-cents, and young adults ≤25 years, a target HbA1c of <7.0% is recommended (Table 2). This low target for HbA1c seems to be associated with better metabolic outcomes [111]. The utility of HbA1c is further enhanced when used in conjunction with glycemic data measured by continuous glucose monitoring (CGM). The ADA published recommenda-tions for time in range (TIR) targets when using CGM. Spending 70% TIR corresponds to a HbA1C of 7.0% [112]. The use of a next-generation hybrid closed-loop insulin delivery system allows improvements in HbA1c and TIR with no increase in hypoglycemia [113].

### 2.3. High Blood Pressure

In T1D, arterial hypertension is a well-recognized risk factor for CVD [119] and diabetes-related complications such as microalbuminuria, diabetic nephropathy, and diabetic retinopathy [120]. Elevated BP during childhood was shown to be a predictor of adult hypertension [121]. Early complications due to hypertension start at an early age. The large, multicenter SEARCH for Diabetes in Youth (SEARCH) study demonstrated that hypertension was linked to arterial stiffness [47] and elevated CIMT [46]. In a study with 24 h ambulatory blood pressure monitoring, loss of nighttime systolic blood pressure (SBP) dipping was associated with an increase in CIMT [122].

In people with T1D, hypertension is more prevalent than in the general population. Results from a retrospective cohort study showed substantially higher prevalence rates of hypertension in T1D children than in their diabetes-free peers. The prevalence reached almost 35% 20 years after the onset of diabetes [123]. The prevalence of hypertension in children with T1D was reported to be between 4 and 16% and did not significantly differ between girls and boys (Table 1). In the SEARCH study, the estimated prevalence of elevated BP in 3691 youths with T1D aged 3 to 17 years was 5.9% [71]. In a study from Norway with 1658 patients, mean age 13 years, 4% of individuals had BP levels > 95th percentile [72]. In a DPV study of >27,000 youths, the prevalence was approximately 10% [70]. Even higher prevalence was found in 1433 Australian children with T1D aged <18 years, where hypertension was found in 16% of the study population [124]. In healthy children, BP may increase more during puberty, and this rise is more pronounced in boys [125]. Tracking of blood pressure revealed that children with elevated BP and T1D had higher BP later in life and that the prevalence of hypertension increased after puberty [126].

There are several distinct pathophysiologic mechanisms that are implicated in the development of hypertension in T1D, including endothelial dysfunction, activation of the sympathetic nervous system, activation of the renin–angiotensin system, and other yet-undefined mechanisms [127]. In adults with T1D, sustained hyperglycemia, as measured by HbA1c level, has been shown to be a risk factor for the development of hypertension. Intensive therapy during the DCCT reduced the risk of incident hypertension by 24% during EDIC study follow-up. The antihypertensive effect of intensive insulin therapy was explained by improved glycemic control. In addition, older age, male sex, family history of hypertension, greater BMI, and albuminuria were independently associated with the development of hypertension [128].

Risk factors for abnormal blood pressure patterns and hypertension in youths with T1D include obesity and hyperglycemia. In the DPV study, ambulatory blood pressure monitoring was performed in 2105 children with T1D aged 5–18 years. In particular, nocturnal BP was significantly elevated in youths with T1D. Insulin dosage, female sex, BMI, HbA1c, and diabetes duration were significantly associated with increased BP. Since obesity is a well-known risk factor for the development of hypertension through insulin resistance, girls might have been predisposed to early blood pressure alterations because of increased weight gain and a higher risk for insulin resistance during puberty [129]. Results from the SEARCH study indicated that waist-to-height ratio as a marker of central obesity may be an important factor for hypertension in youths and young adults with diabetes [130].

Identification and treatment of hypertension in children with T1D is necessary to prevent further micro- and macrovascular complications. Hypertension in children and adolescents is defined as SBP and/or diastolic blood pressure (DBP) that is ≥95th percentile for sex, age, and height on more than three occasions [131]. In children with T1D, the target blood pressure ≤90th percentile for age, sex, and height is recommended by the ADA and ISPAD (Table 2). Children and adolescents with blood pressure levels ≥120/80 mmHg (even if below the 90th percentile) should also be considered prehypertensive [15,110]. Blood pressure should be measured at every visit, and a 24 h ambulatory blood pressure measurement may be helpful for confirmation of hypertension. For prehypertension, initial treatment includes lifestyle interventions to achieve or maintain normal BMI, including diet and physical activity. When hypertension is confirmed, medical therapy, usually with an angiotensin-converting enzyme inhibitor (ACE) or angiotensin receptor blocker (ARB), is recommended [15].

### 2.4. Albuminuria

Diabetic kidney disease (DKD) as well as the presence of albuminuria are strong risk factors for CVD [56,132] and mortality [133,134]. A retrospective study in young T1D population (*n* = 205) with a mean T1D duration 11.32 +/− 4.02 years and mean age at diagnosis 6.10 +/− 3.54 year found that end-stage renal disease occurred in 2.9%, microalbuminuria in 11.2% and proteinuria in 6.8% of its population [75]. In observational SEARCH study the prevalence of diabetic kidney disease in youths with T1D was 5.8% [135]. In a retrospective observational cohort study of T1D children and adolescents the prevalence of abnormal urinary albumin excretion was 9% [136]. Albuminuria was present in 3% of adolescents with only 2 to 5 years of diabetes duration [137]. Data from the Adolescent Type 1 Diabetes Cardiorenal Intervention Trial (AdDIT) trial demonstrated greater age- and sex-adjusted pulse wave velocity and greater aortic intima media thickness in adolescents with T1D with higher albumin excretion [138,139].

Although improved glycemic control is associated with reduced risk of microalbuminuria [51] the risk for DKD is not fully understood. A prospective DPV study of youths with T1D (*n* = 27,805) showed that HbA1c, blood pressure, dyslipidemia, diabetes duration, and male sex were correlated with development of nephropathy [76]. In the SEARCH study female sex, HbA1c and triglyceride values and hypertension were significantly associated with elevated urinary albumin/creatinine ratio (ACR) [73]. Another retrospective study found that there was a significant association between the occurrence of microalbuminuria or proteinuria and poor glucose control as well as higher LDL-c and age greater than 6 years at diagnosis [75]. In the T1D Exchange study microalbuminuria was present in 329 of 7549 (4.4%) participants. Higher frequency was associated with longer diabetes duration, higher mean HbA1c level, older age, female sex, higher diastolic blood pressure and lower BMI [77]. Dietary assessments of 461 youths and young adults with T1D from the SEARCH study showed borderline inverse association between adherence to a higher-quality diet and microalbuminuria, although adherence to healthy diets was low in this cohort [78]. There are discrepancies in the reports on sex differences in diabetic kidney disease in adults. Recent review concluded that men with either T1D or T2D appear to be at higher risk of diabetic kidney disease than premenopausal women. This protection is lost in postmenopausal women and women with concomitant risk factors such as hypertension [140].

Albuminuria can regress with tight glycemic control, and antihypertensive agents [141]. In adolescents in particular albuminuria can spontaneously regress, suggesting it may represent reversible endothelial injury [142]. In a retrospective observational cohort study of T1D children and adolescents the prevalence of abnormal urinary albumin excretion was 9%. However, in 14 of 17 untreated individuals and 79% of ACE–treated patients albuminuria reversed suggesting albuminuria may be reversible in youths with T1D [136]. ADA defines albuminuria (formerly known as microalbuminuria) as an albumin/creatinine ratio of ≥30 mg/g creatinine (Table 2). Levels above this range denote proteinuria (previously known as macroalbuminuria). In the presence of diabetic nephropathy blood pressure should be closely monitored [143]. In youths with T1D annual screening for albuminuria is recommended 2–5 years after diagnosis of T1D once a child reaches 11 years of age. Improved blood pressure control with renin-angiotensin-aldosterone system inhibition is an established method of reducing persistent albuminuria [15]. However, data from the T1D Exchange showed that only 36% of participants with elevated albumin excretion received renin-angiotensin-aldosterone system inhibitors [77].

### 2.5. Dyslipidemia

Dyslipidemia is considered one of the most important CVD risk factors in people with diabetes, though it may be severely undertreated [144]. People with T1D and good metabolic control have similar lipid profiles as the healthy population, but their lipid composition might still be more atherogenic [18,145,146]. On the other hand, suboptimal metabolic control could lead to diabetic dyslipidemia, which is characterized by high levels of LDL-c and triglycerides (TG) and low levels of HDL-c [146,147]. The longitudinal EDC study showed that more than ten years of elevated LDL-c levels and lower HDL-c levels are associated with a higher risk of CVD in a T1D population [148]. Diabetes is considered as a high-risk condition for accelerated atherosclerosis development, requiring regular lipid monitoring and early intervention [149]. Failure to diagnose dyslipidemia in children may at least in some cases preclude an opportunity to prevent the long-term consequences of CVD, which is among the leading causes of morbidity and mortality and generates substantial medical costs in the developed world [150,151].

The National Heart, Lung, and Blood Institute (NHLBI) guidelines recommend targeted screening of those most likely to develop early CVD. This screening should be performed in children aged 2 to 8 years with diabetes and some other conditions [114]. According to ADA guidelines, T1D children ≥2 years of age should have a fasting lipid profile soon after diagnosis and after achieving glucose control. If initial LDL cholesterol is ≤2.6 mmol/L, subsequent testing should be performed at 9–11 years of age. If lipid screening is normal, screening is recommended every two to three years [15,110] (Table 2). Stringent lipid goals are thus already set for children and adolescents with diabetes by ISPAD and ADA guidelines, which, to reduce CVD risk, recommend LDL-c and TG levels to be below 2.6 and 1.7 mmol/L, respectively, and HDL-c levels to be above 1.1 mmol/L [152,153]. However, many children with T1D are still above the goals. A study by Macedoni et al. showed that 26.3% of 467 participants had LDL-c levels above the type 1 diabetes goal of 2.6 mmol/L, while 3.6% had LDL-c levels above 3.4 mmol/L and 1.1% above 4.1 mmol/L [6]; in a study by Reh et al., 50% of the study population had LDL-c levels above 2.6 mmol/L at baseline, while during the 3-year follow up, this figure rose to 58% [154]. Similar figures were also reported by Kershnar et al. [155].

Lipid levels in children vary with age and sex. Lipid levels are relatively stable from the age of two until puberty (for most children until 9–11 years of age). During puberty, TC and LDL-c levels decrease by 10–20% and rise again in the late adolescence. Lipid levels in childhood are predictive of lipid levels in adulthood, with the highest correlation between late childhood and the third to fourth decades of life [151]. In girls, the median Tc levels are higher than in boys [156,157]. Boys experience a decrease in high-density lipoprotein HDL-c levels during late puberty, whereas HDL–c levels remain stable in girls until menopause [114].

Young females with T1DM had higher mean TC, LDL-c, non-HDL-c, and ApoB (but also HDL-c) levels than males, despite the fact that their HbA1c levels were no different from those of males, implying a less favorable CVD risk profile in women with T1D even at younger ages [6] (Table 1).

### 2.6. Obesity and Insulin Resistance

Registry data indicate that youths with T1D have a higher prevalence of overweight or obese status compared with healthy peers, with unhealthy body weight affecting a substantial proportion of individuals with T1D [158,159,160]. Several studies have directly compared individuals with T1D to healthy populations of the same time period matched for age and sex [161]. The SEARCH for Diabetes in Youth Study found that youths with T1D (*n* = 3524) had significantly higher prevalence of overweight status (22.1%) compared with youths without T1D from the general population reference sample (16.1%) [79]. In a DPV study, predictors of weight gain among 12,774 children and adolescents with T1D were analyzed. Of youths with T1D, 12.5% were overweight and 2.8% were obese. Female sex, pubertal diabetes onset, long diabetes duration, lower BMI-standard deviation score at diabetes onset, intensive insulin therapy, and higher insulin dose were significant predictors of weight gain [81]. Youths 2 to 18 years of age diagnosed with T1D for at least 1 year from two large clinical registries (the Type 1 Diabetes Exchange and DPV) had greater BMI z-scores compared with respective national reference samples [158]. Cross-sectional data collected from 308 children aged 3–17 years and 283 young adults aged 18–30 years with T1D in Australia showed that overweight and obesity rates were, when compared with the matched population, both significantly higher in the T1D population aged 5–16 years [85]. In addition, longitudinal persistence of overweight status and obesity was observed for a large proportion of 11,513 youths with T1D across three large registries (T1D Exchange, DPV, and the Australasian Diabetes Data Network (ADDN)), suggesting that individuals who have overweight status or obesity at puberty will continue to have overweight status or obesity as adults. The rates of obesity at the end of the follow-up period ranged from 9 to 18% [86]. Obesity is also prevalent among adults with T1D. The EDC study of temporal trends in adults with T1D showed an increase in overweight status and obesity, reaching 42% and 22.7% after 18 years of follow-up. This was in parallel with a nearly 10-fold increase in the prevalence of intensive insulin therapy. In addition to intensive insulin therapy, higher baseline HbA1c was a predictor of weight gain. The rise of prevalence of overweight status and obesity occurred faster than in the general population and was therefore not due to aging of the cohort, and it was similar in men and women [162]. On the other hand, a study with 507 youths (age 8–16 years) with T1D from four separate cohorts showed that the prevalence of overweight status or obesity was 33% and remained stable over a decade despite increased implementation of intensive insulin therapy [163].

Treatment regimen is a specific factor associated with weight gain in the T1D population. Modern intensive insulin therapy either with MDI or CSII, which was introduced into clinical practice to achieve glycemic targets, has a recognized association with weight gain compared with conventional therapy. DCCT was one of the earliest studies demonstrating the association of intensive insulin therapy with weight gain and obesity [164]. However, there is no general consensus as to whether there are differences in weight gain between CSII and MDI. A more recent DPV study of 5665 youths with T1D showed that a minor association of intensified insulin therapy with BMI and CSII use was related solely to a higher risk of belonging to the increasing BMI z-score trajectories [84]. In a study comparing longitudinal trajectories of BMI z-score from childhood to adolescence across three registries (T1D Exchange, DPV, and ADDN), insulin pump therapy was more common in the T1D Exchange and ADDN than in the DPV, and it was not associated with membership to higher BMI z-score trajectories [86]. It is yet uncertain what effect closed-loop systems that may potentially allow even tighter control will have on obesity in people with T1D [161].

There is a sex-related difference regarding overweight status and obesity in T1D (Table 1). Several studies showed high rates of overweight status and obesity in girls [79,82,85]. During the 6 years following diagnosis of T1D in 209 pediatric patients, an increase of BMI z-score was more pronounced in girls [80]. Another study showed excessive weight gain in girls with longer diabetes duration [81]. The longitudinal study over 13 years from the DPV database revealed substantial differences between girls and boys [84]. Girls were more likely to be in the increasing BMI z-score, stable high BMI z-score, or chronically overweight groups, whereas boys were more often in the low stable or decreasing BMI z-score classes. Similar results were found in an international study of BMI trajectories by sex, with girls more often experiencing pubertal weight gain [86]. The reasons for sex-related differences in overweight and obesity rates in youths with T1D are not well understood and might be partly explained through differential hormonal changes, body composition, and energy metabolism during puberty in males and females [83]. In line with this, even in healthy pubertal girls, insulin resistance was more pronounced [165]. In addition, the higher rates of overweight status and obesity in teenage girls than in boys might be partially explained by psychosocial factors such as higher psychological burden of the diagnosis of diabetes [166].

In T1D, overweight status and obesity are substantial risk factors for macrovascular and microvascular complications [167]. Excessive weight gain in DCCT was associated with sustained increases in lipid levels and blood pressure as well as with more extensive atherosclerosis during EDIC [168]. In adults with T1D, obesity was significantly associated with an increased risk for cardiovascular disease and retinopathy despite similar HbA1c compared to nonobese individuals with T1D [169]. Moreover, elevated body weight can result in insulin resistance and metabolic syndrome [167]. The metabolic syndrome criteria include visceral obesity, atherogenic dyslipidemia, and hypertension [170]. The presence of metabolic syndrome components in T1D is associated with an increased incidence of chronic complications and mortality later in life [171]. Among children, adolescents, and young adults with T1D, the prevalence of metabolic syndrome was significantly higher among overweight (8.1%) and obese (35.3%) individuals than among normal weight (4.9%) individuals with T1D [83]. A higher BMI was related to worse metabolic control [84] as well as higher rates of severe hypoglycemia in youths with T1D [158]. In youths and young adults with T1D, BMI-SDS was strongly associated with dyslipidemia and hypertension and a high proportion of overweight and obese youths had multiple CVD risk factors [85]. In addition, BMI z-score was the only modifiable CVD risk factor that predicted CIMT youths with T1D [172].

The American Medical Association (AMA)/Centers for Disease Control and Prevention (CDC)/Maternal and Child Health Bureau (MCHB) expert committee defined a BMI at or greater than the 95th percentile as obese and a BMI between the 85th and 94th percentiles as overweight [173]. In overweight and obese youths, lifestyle modifications that include a healthy diet, regular physical activity, and reduced sedentary activity should be initiated to target a normal BMI. However, only a small number of weight-loss and lifestyle interventions for obesity in children and adolescents have been shown to be effective in primary care settings [114]. In adults with T1D, the REducing with MetfOrmin Vascular Adverse Lesions in Type 1 Diabetes (REMOVAL) study demonstrated that 3 years of metformin therapy reduced atherosclerosis progression measured by averaged maximal CIMT, despite no improvement in glycemic control [174]. The Metformin Therapy for Overweight Adolescents with Type 1 Diabetes study in overweight adolescents with T1D demonstrated reduction in insulin dose and measures of adiposity, although no improvements in glycemic control were observed [175]. In youths with T1D between the ages of 8 and 18 years, metformin improved vascular smooth muscle function and glycemic control and lowered insulin dose [176]. Similarly, 3-month metformin therapy in youths with T1D between the ages of 12 and 21 years reduced insulin resistance as measured by hyperinsulinemic euglycemic clamp. In addition, improvements in BMI, fat mass, and measures of aortic and carotid vascular health were observed [177].

### 2.7. Chronic Inflammation

Several prospective studies have highlighted the association between basal C-reactive protein (CRP) concentrations and future cardiovascular events in young individuals with no CVD [178]. High-sensitivity CRP (hsCRP) was added to the traditional risk factor screening included in the Framingham Risk Score as an independent prognostic inflammation marker for CVD in the adult population [115]. A meta-analysis showed that hsCRP is a more accurate prognostic factor for CVD than increased arterial blood pressure or cholesterol concentration [179]. On the other hand, a systematic review of 31 prospective cohort studies found that, overall, CRP did not perform better than the Framingham risk equation, and that the improvement of risk stratification through the addition of CRP to established predictive models was small and inconsistent [180]. The 2016 European guidelines on cardiovascular disease prevention state that, while hsCRP has shown consistency across large prospective studies as a risk factor integrating multiple metabolic and low-grade inflammatory factors, it is not advised to routinely assess circulating biomarkers (including hsCRP) for refinement of CVD risk assessment (recommendation class III level B), especially in patients with clearly high or low risk [181]. Several studies also linked hsCRP value to metabolic syndrome and the development of type 2 diabetes (T2D) incidence and found that statins and anti-inflammatory agents may reduce cardiovascular events in direct proportion to hsCRP levels [182].

Scarce data exist on the use of the hsCRP as prognostic inflammation marker for CVD and on its relationship with the other traditional cardiovascular risk factors in young people with T1D [110,115]. Inflammation status and oxidative stress have been recently described as possible triggers of the progression to a clinically evident CVD in T1D subjects. After diabetes onset, a chronic state of low-grade inflammation seems to persist in subjects with T1D. Hyperglycemic fluctuations may then magnify the inflammation, increasing the levels of inflammation markers in subjects with diabetes compared with healthy people [183].

In particular, in women, hsCRP was found to be a stronger predictor of cardiovascular events than LDL-c [184,185]. The higher prevalence of low-grade inflammation in females has been extensively described in adults [186,187]. However, few data exist on pediatric youths with T1D (Table 1). In a study by McKenzie et al. on 124 children and adolescents with T1D, hsCRP was significantly higher in females compared to males, when controlled for BMI [87]. Moreover, when healthy controls matched by age were considered, hsCRP was still higher in females compared to males and approached significance. In a study by Brown et al. including 300 subjects with T1D, hsCRP was significantly higher in females than in males when adjusted by pubertal stage, ethnicity, and smoking status [20].

### 2.8. Lifestyle, Diet and Psychosocial Factors

#### 2.8.1. Physical Inactivity

Sedentary lifestyle and physical inactivity are among the leading modifiable risk factors for future CVD [188]. Moy et al. showed that activity level is inversely associated with mortality risk [189]. Tikkanen-Dolenc et al. showed that exercise, particularly high-frequency and high-intensity exercise, may reduce CVD risk in individuals with type 1 diabetes [190].

Randomized controlled trials have shown the beneficial effect of physical activity on CVD risk profile in youths with T1D. In a DPV cross-sectional study (*n* = 23,251, age 3–18 years), increased frequency of regular physical activity (exercise once a week for 30 min) was strongly associated with improved glycemic control as well as lower diastolic blood pressure and lipoprotein levels [191]. In addition, a DPV cross-sectional study of adults showed an inverse correlation between physical activity and HbA1c, diabetic ketoacidosis, BMI, dyslipidemia, hypertension, and retinopathy or microalbuminuria without an increase of adverse events [192]. In adolescents with T1D, exercise improved glycemic control and dyslipidemia as well as reducing cardiovascular risk factors such as BMI and waist circumference [193]. Furthermore, exercise capacity was strongly associated with renal health, which is an important determinant of CVD in adults with T1D [194]. Meta-analysis of physical activity intervention studies in children and young people with T1D identified potential benefits of physical activity on reducing HbA1c, BMI, TG, and Tc [195]. A systematic review of physical activity interventions in youths with T1D (<18 years) showed a reduction of HbA1c, indicating an improvement in glycemic control. Findings suggested that longer interventions (program duration >12 weeks), more frequent activity (≥3 sessions/week), longer activity duration (≥60 min/session), and inclusion of resistance exercise together with aerobic activity may have been most effective at improving HbA1c. However none of the included studies targeted sedentary behavior [196].

Youths with T1D of both sexes had, on average, reduced physical fitness levels compared to their nondiabetic peers. Female gender, increasing age, higher skinfold thickness, lower physical activity level, and poor long-term metabolic control were significant independent predictors of lower maximal aerobic capacity (VO2 max) as a measure of cardiorespiratory fitness [90]. De Lima et al. showed that teenagers with T1D had less physical activity and less cardiorespiratory capacity than healthy controls. Teenagers who dedicated a greater time in moderate-to-vigorous-intensity physical activity demonstrated better glycemic control [197].

Some data suggest sex-specific differences in physical activity in youths with T1D (Table 1). Adolescent girls with T1D were found to be less active than boys [88,89]. Only 5% of 203 adolescent girls with TID met international recommendations by reporting exercising 60 min a day, 7 days per week. On average, girls reported being physically active three days per week for at least 60 min [198]. On the other hand, analysis published on a cohort of 211 adolescents with T1D reported almost equivalent physical activity between girls and boys [91].

The fear of hypoglycemia may be an important barrier to regular physical activity in youths with T1D [199]. ISPAD recommends regular physical activity in order to avoid micro- and macrovascular complications [15]. Children and adolescents with T1D should be encouraged to engage in at least one hour or more of physical activity each day (Table 2). Barriers to physical activity should be addressed, and education on blood glucose management in exercise should be offered [116].

#### 2.8.2. Unhealthy Diet

Nutrition and eating behavior are important factors to consider with regards to T1D management. Poor dietary habits increase the risk for early microvascular and macrovascular complications [200]. However, it has been shown that youths with T1D tend not to achieve dietary recommendations. In a large cohort of 1697 youths with either T1D or T2D of at least 1-year duration, dietary habits were poorer than in the general population. Patterns of adherence were similar for boys and girls. Fewer than 15% of youths within each age and diabetes type subgroup met the ADA and American Heart Association (AHA) recommendations for total and saturated fat intake. Intake of fruits, vegetables, and grains was similarly inadequate [94]. In a cross-sectional study of food habits of Swedish adolescents with T1D, the intake of protein in boys was higher and the intake of fiber in girls were lower than the calculated recommendations. The intake of saturated fat was higher and the intake of polyunsaturated fat was lower than recommended in both boys and girls. Snacks contributed to as much as 30% of total daily energy intake for girls. In general, adolescents with T1D had healthier food habits than healthy control subjects [201]. Analysis of dietary intake in 655 young people with T1D (3 to 19 years of age) showed that skipping meals was associated with negative characteristics such as suboptimal HbA1c and LDL-c levels, watching more TV, being overweight, and having a higher intake of added sugar and lower intake of fiber compared with those who do not skip meals. Those who skipped meals were more likely to be girls than boys [95]. Analysis of eating patterns in 104 adolescents showed that a significantly lower proportion of females with T1D (73.8%) than males (97.7%) consumed breakfast on a daily basis, and this was significantly associated with poor metabolic control among females [96] (Table 1).

Dietary recommendations for children with T1D are based on healthy eating recommendations for the general population, which, along with healthful eating, should provide energy intake and nutrients for normal growth and development. According to the ISPAD guidelines for children and adolescents with T1D, carbohydrates should account for approximately 45% to 50% of energy, fat for <35% (saturated fat < 10%), and protein for 15% to 20% of energy [117] (Table 2). However, it remains unclear which of the macronutrient proportions are optimal for daily glycemic control and diabetes-related outcomes.

#### 2.8.3. Eating Disorders

Because of the increased focus on diet and weight gain associated with insulin treatment, there may be an increased risk for the development of comorbid disturbed eating behaviors and eating disorders in T1D [202]. Three main types of eating disorders are anorexia nervosa, bulimia nervosa, and binge eating disorder [203]. Disordered eating behaviors refer to problematic eating behaviors such as food restriction for weight control, binge eating, and purging behaviors that do not yet meet the clinical criteria for the diagnosis of an eating disorder [204]. Insulin restriction (reducing or omitting insulin) is diabetes-specific disordered eating behavior of controlling weight by purposeful underdosing or complete omission of the required insulin in order to excrete glucose through the urine (purge calories via glucosuria) [205]. Some studies have shown higher prevalence of disturbed eating behaviors among individuals with T1D compared to individuals without diabetes. A systematic review indicated that the prevalence of disordered eating behavior was 39.3% in adolescents and young adults with T1D and 32.5% in peers without T1D. The prevalence of eating disorders was 7.0% in the T1D population and 2.8% in nondiabetic peers [206]. In the SEARCH study, disturbed eating behaviors were observed in 21.2% of 2,156 youths and young adults with T1D who were 10–25 years old (mean ± SD age 17.7 ± 4.3 years). The prevalence of disturbed eating behaviors was highest in the 15- to 19-year-old age group, indicating that adolescence is a critical period of risk for the onset of disturbed eating behaviors [99].

As in the general population, the prevalence of disturbed eating behaviors in adolescent and young adult females with T1D is higher than in males (Table 1). Among 770 children and adolescents 11–19 years of age with T1D from the Norwegian Childhood Diabetes Registry, 27.7% of females and 9% of males with T1D scored above the cutoff for disturbed eating behavior. In addition, 31.6% of the participants admitted to restricting insulin after overeating [97]. Similarly, 31.2% of 289 11- to 17-year-old females and 11.7% of 340 males with early onset of T1D from a German nationwide, population-based cohort study scored positive for symptoms of disturbed eating behaviors [98]. In the SEARCH study, among those who had disturbed eating behaviors, approximately 71.2% were female [99]. In a longitudinal study over a 14-year period beginning in late childhood, eating disorders were common and persistent. At study termination, 32.4% of participants met the criteria for a current eating disorder, and an additional 8.5% had a subthreshold eating disorder. Insulin omission as a weight control method was reported by 27% of participants [207]. Less frequent breakfast consumption among female adolescents with T1D was associated with increased eating disorder pathology [208]. Similarly higher BMI and older age significantly correlated with disturbed eating behaviors [99,208,209].

Disordered eating behaviors have been associated with poorer glycemic control [97,99,206] and more frequent diabetic ketoacidosis episodes [99] in adolescents and in young adults with T1D. Poor metabolic control may lead to long-term diabetes-related complications [210], and insulin restriction is associated with a threefold increase in mortality rates [211]. Screening [209] and attention to the potential warning signs of disturbed eating behaviors in adolescents with diabetes and subsequent early intervention, which should include a multidisciplinary approach, may help to improve long-term outcomes of this comorbidity [110] (Table 2).

#### 2.8.4. Smoking

Smoking is among the leading preventable causes of morbidity and mortality and a major independent risk factor for CVD in individuals with diabetes mellitus [212,213]. Approximately 90% of adult smokers report having started smoking before 18 years of age [214]. Because of the already increased risk of CVD in individuals with diabetes, the ADA emphasizes the importance of smoking cessation in people with diabetes [213]. Reynolds et al. that reported the prevalence of tobacco use in youths aged 10 to 14 years, 15 to 19 years, and ≥20 years with T1D was 2.7%, 17.1%, and 34.0%, respectively. Fewer than 50% of youths aged 10 to 14 years (52.2% of participants) reported having ever been counseled by their healthcare provider not to smoke or to stop smoking [93]. It is urgent both to regularly ask about smoking habits and to educate on the severe consequences of smoking for subjects with T1D in order to prevent adolescents from smoking [215].

In a study of 27,561 patients aged 15 to 20 years with T1D, Hofer et al. found that self-reported cigarette smoking (one or more cigarettes per day) was practiced by 21.6% of male and 13.7% of female participants; smokers had a worse cardiovascular risk profile than nonsmokers [92]. Reynolds et al. reported that among 2887 participants aged 10–22 years, 11% males and 8.8% females used tobacco products [93]. Thus, possibly somewhat more male adolescents with T1D might smoke as compared to females (Table 1).

#### 2.8.5. Psychosocial Factors

Symptoms of depression, anxiety, and psychological distress tend to be common in young people with T1D [216]. Among 2672 youths from the SEARCH study (aged 10–21 years), 14% had mildly and 8.6% had moderately or severely depressed mood. Girls had reported more depressed symptoms than boys [100] (Table 1). In the Pediatric Diabetes Consortium Screening Assessment of Depression in Diabetes study, subclinical depressive symptomatology was identified in 13% of 261 T1D participants aged 10 to 18 years, and only 4% of these were treated by a therapist within the prior 12 months [101]. Anxiety symptoms were present in 17% of adolescents and were associated with less frequent blood glucose monitoring and suboptimal glycemic control in adolescents with T1D [217]. Diabetes distress refers to the emotional burden and worries that arise from living with and managing diabetes. A systematic review revealed that around one-third of adolescents with T1D experienced diabetes distress with no consistent relationship with gender [102]. How individuals cope with diabetes-related stress may mediate diabetes self-management and effect glycemic control. Adolescents with T1D appear to use avoidant coping strategies such as avoiding or delaying of problem-solving, disengagement from emotions and thoughts related to the problem, and denial of the immediacy or seriousness of a problem. These strategies have been linked with reduced adherence and poorer glycemic control [218].

Depressive symptoms were associated with higher HbA1c_,_ less frequent blood glucose monitoring, and increased risk of hospitalization in young people with T1D [219]. Comorbid depression represents an important risk factor in the development of coronary heart disease (CHD) in adults with T1D [220]. An EDC study of 658 men and women with T1D demonstrated that participants who reported the fewest depressive symptoms on the Beck Depression Inventory had the least risk of developing CHD over a 10-year period. Gender did not appear to moderate the strength of the relationship between depressive symptomatology and any CHD endpoints [221]. However, in an earlier EDC study, depression was a strong predictive risk factor for coronary artery disease in women [222].

Health-related quality of life (HRQL) is an important outcome in diabetes care. In 910 participants with T1D from a longitudinal observational SEARCH study, girls reported significantly worse emotional, school, social functioning, and total HRQL as they grew older than boys, who had stable or significantly improved HRQL as they aged. HRQL scores were related to greater parent education, lower HbA1c values, and greater physical activity [103]. Therefore, lower HRQL scores in adolescent girls with T1D may suggest greater risk for poor glycemic control. In a prospective study sample of economically disadvantaged children with T1D, high family cohesion predicted significantly better glycemic control several years later, but only among girls. This finding suggests that supportive cohesive families may be particularly protective for girls [223].

The ADA and ISPAD recommend regular screening for psychosocial status, impact of diabetes on quality of life, diabetes distress, symptoms of anxiety, disordered eating behaviors, eating disorders, and symptoms of depression (Table 2) [110,118]. Several family-based behavioral interventions, usually delivered by mental health professionals at the time of outpatient medical clinic appointments, have demonstrated improved adherence and glycemic control [224]. In an observational study using the DPV database, youths with comorbid T1D and depression who were treated with antidepressants had lower HbA1c and showed a statistically nonsignificant trend towards higher BMI compared to nontreated depressed youths [225].

## 3. Sex and Cumulative Number of Risk Factors

Having multiple CVD risk factors substantially increases the risk for CVD and mortality [226]. A higher cumulative number of CVD risk factors was associated with more progressive early atherosclerotic lesions [227]. A higher prevalence of several CVD risk factors [70,72,87,147] and early microvascular complications [74,228] has been reported for adolescent girls compared with boys. Cardiovascular risk factors such as obesity, hypertension, dyslipidemia, poor glycemic control, and smoking were analyzed in 27,358 prepubertal, pubertal, and young adult participants with T1D from the DPV database. The prevalence of elevated HbA1c, BMI, Tc, and LDL-c was significantly higher in women than in men. On the other hand, frequency of hypertension and current smoking was significantly higher in men than in women. Of the participants, 16% had two or more risk factors [70]. In a subsequent DPV study, 33,488 children and adolescents ≤ 18 years were sorted into five categories by their number of CVD risk factors. Dyslipidemia (Tc *>* 5.17 mmol/L, HDL-c *<* 0.91 mmol/L, and/or LDL-c *>* 3.36 mmol/L), elevated systolic and/or diastolic BP ≥ 90th percentile, BMI *>* 97th percentile, active smoking, and HbA1c ≥7.5% were considered as CVD risk factors. Of all participants, 65% had zero or one CVD risk factors and 35% had two or more CVD risk factors. Except for smoking and HDL-c, the proportion of girls per CVD factor was higher for the majority of CVD risk factors compared to that of boys. The differences were more pronounced for dyslipidemia and elevated BP. The proportion of girls with no CVD risk factors was 45%, but that in the categories with 4 to 5 CVD risk factors was 60% [147]. Among children and adolescents with T1D in Norway (mean age 13.1 years), 86% had at least one of the risk factors for CVD, 45% had two or more risk factors, 15% three or more risk factors, and 2% four or more risk factors. CVD risk factors included HbA1c above the target level; positive family history of early CVD and/or diabetes; LDL-c > 2.6 mmol/L; HDL-c < 1.1 mmol/L; BP above the 90th percentile by age, sex, and height; BMI above the 95th percentile; smoking; and persistent microalbuminuria. Differences in prevalence per risk factor between boys and girls were reported only for LDL-c and HDL-c. A higher proportion of girls had elevated LDL-c, and a higher proportion of boys had lower HDL-c [72]. Results from a single-center study demonstrated that adolescent girls with T1D had significantly increased mean HbA1c levels, BMI, LDL-c, and C-reactive protein and lower systolic BP compared with boys with the disease. Physical activity was equivalent among the study participants [20].

## 4. Conclusions

CVD is the leading cause of morbidity and mortality, severely increasing the early mortality rates in persons with T1D as compared to healthy individuals [1,4]. In addition, the mortality ratio for CVD has been reported to be 40-fold in women with T1D before the age of 40 years, compared to tenfold increase in men with T1D, implying a more atherogenic profile in young women with T1D [5,18,19,20]. In this review, we explore the role of CVD risk factors in children and adolescents with T1D, with particular regard to sex-related differences in risk profile.

Based on the extensive review of the existing literature, we found a clear difference between boys and girls with T1D in the presence of individual CVD risk factors as well as in their overall CVD risk profiles (please see the Table 1 for a summary presentation). Girls were, on the whole, more impacted by the major risk factors (hyperglycemia, dyslipidemia, obesity, inflammation, physical inactivity, unhealthy diets) than boys. In addition, girls tended to have more risk factors at the same time than boys. This might be related to the less favorable CVD morbidity and mortality outcomes in females with T1D as compared to that in males. We strongly argue for early CVD prevention in people with T1D, which should start in the pediatric age and be tailored to address sex-related differences.

Pediatric guidelines for early intervention addressing CVD risk in T1D typically rest on insufficient prospective data. Further studies are needed to specifically address the role of sex-related differences in CVD risk profile and in early CVD prevention. Existing clinical registries could play important role to address this issue in the future. In addition, introducing noninvasive imaging technology may also help identify patients who are at particularly high risk early with possible evidence of end-organ damage [9,229,230].

To conclude, early identification of CVD risk factors and possibly also sex-specific intervention would have the potential to reduce later CVD morbidity and excess mortality in females with T1D. Further research and clearer clinical guidance are needed to better address this issue.

## Figures and Tables

**Table 1:** Types of cardiovascular risk factors and their relation to sex in children and adolescents with type 1 diabetes.

Type of CVD Risk Factor	CVD Risk Factor	Sex-Related	Sex with Higher Overall Burden (Degree) ^1^	Study (Sex Difference Girls vs. Boys)
Nonmodifiable	Age/duration of T1D	No	NA	NA
	Family history of CVD	No	NA	NA
	Ethnicity	No	NA	NA
	Genetic risk factors	No	NA	NA
**Modifiable**	Hyperglycemia	Yes	Girls (++)	Schwab 2006 [70] (Girls) Gerstl 2007 [61] (Girls) Hanberger 2013 [65] (Girls) Dovc 2014 [64] Girls (Girls) McKnight 2015 [68] (Girls) Brown 2016 [20] (Girls) Samuelsson 2016 [66] (Girls) Carlsen 2016 [67] (Girls) Maiorino 2018 [69] (Girls)
	High blood pressure	Unclear	NA	Rodriguez 2010 [71] (No difference) Margeirsdottir [72] 2008 (No difference) Schwab 2006 [70] (Boys)
	Albuminuria	Unclear	NA	Maahs 2007 [73] (Girls) Margeirsdottir 2008 [72] (No difference) Amin 2008 [74] (Girls) Salgado 2010 [75] (No difference) Raile 2007 [76] (Boys) Daniels [77] 2013 (Girls) Costacou 2018 [78] (No difference)
	Dyslipidemia	Yes	Girls (+)	Schwab 2006 [70] (Girls) Margeirsdottir 2008 [72] (Girls) Macedoni 2018 [6] (Girls)
	Obesity	Yes	Girls (++)	Liu 2010 [79] (Girls) de Vries [80] 2013 (Girls) Frohlich-Reiterer [81] 2014 (Girls) Szadkowska [82] 2015 (Girls) Pinhas-Hamiel [83] 2015 (Girls) Prinz 2018 [84] (Girls) Marlow [85] 2019 (Girls) Phelan 2019 [86] (Girls)
	Chronic inflammation	Yes	Girls (+++)	MacKenzie 2009 [87] (Girls) Brown 2016 [20] (Girls)
**Lifestyle and** **Psychosocial**	Physical inactivity	Yes	Girls (+)	Valerio 2007 [88] (Girls) Aman 2009 [89] (Girls) Lukacs 2012 [90] (Girls) Bishop 2014 [91] (No difference)
	Smoking	Yes	Boys (++)	Hofer 2009 [92] (Boys) Reynolds 2011 [93] (Boys)
	Unhealthy diets/eating disorders	Yes	Girls (++)	Mayer-Davis 2006 [94] (No difference) Øverby 2008 [95] (Girls) Wisting 2017 [96] (Girls) Wisting 2013 [97] (Girls) Baechle 2013 [98] (Girls) Nip 2019 [99] (Girls)
	Unfavorable psychosocial factors	Yes	Girls (+)	Lawrence 2006 [100] (Girls) Silverstein 2015 [101] (No difference) Hagger 2016 [102] (No difference) Naughton 2014 [103] (Girls)

CVD, cardiovascular disease; T1D, type 1 diabetes; NA, not applicable.; ^1^ Degree of relation between sex and CVD risk factor: +, mild relation; ++, moderate relation; +++ severe relation.

**Table 2:** Current clinical guidelines for early screening and management of cardiovascular risk factors in children and adolescents with type 1 diabetes.

Type of CVD Risk Factor	CVD Risk Factor	Screening Timing	Target	Management
**Modifiable**	Hyperglycemia and glucose variability	Quarterly HbA1c [109] TIR if possible [110]	HbA1c < 7% without significant hypoglycemia [109,110] TIR (3.9–10.0 mmol/L) > 70% of readings and time below range (<3.9 mmol/L) <4% of readings [112]	Intensified glucose monitoring Use of CGM Use of isCGM Intensified insulin adjustments Use of hybrid closed-loop system [109,110]
	High blood pressure	At least annually [15] At each routine visit [110]	BP < 90th percentile by age, sex and height, <120/80 ≥ 13 years [110]	BP > 90th percentile: Lifestyle intervention, ACE or ARB if BP is still elevated, if microalbuminuria is present [15] BP > 95th percentile: lifestyle intervention and ACE or ARB [15]
	Albuminuria	At 11 years with 2–5 year diabetes duration [15]	ACR < 30 mg/g [110]	ACE or ARB when ACR ≥ 30 mg/g is documented (two of three urine samples obtained over a 6-month interval following efforts to improve glycemic control and normalize blood pressure) [15,110]
	Dyslipidemia	At ≥2 years [110] At ≥11 years [15]	LDL-c < 2.6 mmol/L [15,110] HDL-c >1.2 mmol/L [114] TG < 0.8 mmol/L for children < 9 years of age and <1 mmol/L if >9 years of age [114]	LDL-c ≥ 2.6 mmol/L: lifestyle intervention, optimize glycemic control LDL-c ≥ 3.4 mmol/L: statins if the above interventions fail [15]
	Obesity	Each visit	BMI < 85th percentile for age/gender [114]	Dietary changes and exercise Insulin sensitizing medications
	Chronic inflammation	Unclear	hsCRP ≤ 1 mg/dL [115]	Optimizing glycemic control, BMI, lipid profile, BP
**Lifestyle and** **Psychosocial**	Physical inactivity	Each visit	At least 1h of physical activity daily and minimize sedentary activity [110,116]	Lifestyle intervention
	Smoking	Each visit	None	Education [110]
	Unhealthy diet	At diagnosis, with annual updates [110]	Maintain ideal body weight, optimize growth and development Macronutrient distribution: carbohydrate 45% to 50% of energy (normal daily activity), fat < 35% of energy, saturated fat < 10% of energy, protein 15% to 20% of energy [117]	Nutritional education [110,117]
	Eating disorders	Screening for eating disorders between 10 and 12 years of age [110]	None	Behavioral intervention
	Unfavorable psychosocial factors	At diagnosis and planned intervals [118] Diabetes-related distress from 7–8 years of age [110]	None	Psychosocial interventions Behavioral interventions Antidepressant medications

CVD, cardiovascular disease; T1D, type 1 diabetes; HbA1c, glycated hemoglobin; TIR, time in range; CGM, continuous glucose monitoring; isCGM, intermittently scanned continuous glucose monitoring; BP, blood pressure; ACE, angiotensin converting enzyme inhibitor; ARB, angiotensin receptor blocker; ACR, urinary albumin/creatinine ratio; LDL-c, low-density lipoprotein cholesterol; HDL-c, high-density lipoprotein cholesterol; TG, triglycerides; BMI, body mass index; hsCRP, high-sensitivity C-reactive protein.

## Data Availability

Details of data availability are available from the first author on request.
